# Supplementary material for: Development of protective equipment for endoscopic treatment and surgery in otorhinolaryngology
Source: PLoS One. 2022 Jun 10;17(6):e0268974. doi: 10.1371/journal.pone.0268974 (PMC9187069; doi:10.1371/journal.pone.0268974)
Supplement: Attachment — Submitted filename: Response to reviewers.docx [file pone.0268974.s001.docx]

**Response to Reviewers’ Comments**

Reviewer #1: This study is indeed essential and relevant to be carried out. The paper was well written with acceptable figures. My only concern is that the actual p values were not stated, either in the text or figure legends. Additionally, since the sample size was small, including an **effect size analysis** is beneficial to support the p values.

**Reply:**

Thank you for your encouraging comment.

I have added p-values to the legend of Fig.4 and p-values and the effect sizes in Table 1 in the revised manuscript (lines 243-256).

Reviewer #2: This manuscript is the study to validate the protective equipment for endoscopic 'treatment and surgery'. The simulated situations are droplet spread and aerosol simulation model. The protective equipments are: suction-capable mask, sleeves and cover for suction. The main result is reduction of droplet and aerosol diffusion from there protective equipment.

There are some issues needed to be elucidated.

1) The main result has been displayed in two bar graphs but not in the table. The actual parameter/value will be easier and clearer for verification of 95% Confidence interval and p-value.

**Reply:**

Thank you for your comment.

I have added p-values to the legend of Fig.4. I have also added 95% confidence interval values and p-values as well as the effect sizes to Table 1 in the revised manuscript (lines 243-256)

2) The author mention about the number of test as 'five times' but these values have not been shown in the figures.

**Reply:**

Thank you for pointing this out.

I have indicated the number of tests as “n=5” in the revised version of Fig. 4 and in Table 1 (lines 243-245). I have also added it to the legend of Fig. 4 (lines 246-256).

3) The title mention about 'endoscopic treatment and surgery' but the testing situation was conducted by 'flexible endoscope' which unusual for 'surgery' in ENT.

**Reply:**

Thank you for your constructive feedback.

During surgery, a rigid scope is used. Since the patient is anesthetized during surgery, the risk of splashing due to the patient sneezing, etc., is low. Therefore, the insertion sleeve need not be attached to the rigid scope or treatment tool, and only the surgical mask should be used.

During outpatient treatments, in addition to the outpatient mask, the insertion sleeve is attached to the flexible endoscope or the rigid endoscope and used in combination to reduce the risk of splashing.

In the experiment, the surgical scene (Nos. 1 and 2 in Fig. 4) and the outpatient scene (Nos. 3-6 in Fig. 4) are shown. The flexible endoscope is used only for evaluation in outpatient situations. This content is described in the Experiments section on (lines 145-156).

4) The displayed pictures are difficult for reader to follow the method. More simplified, eg cartoon/drawing, should be done for clarification.

**Reply:**

Thank you for this valuable suggestion.

Cartoon drawings have been added to Fig. 2 to help the readers understand the outpatient and surgical procedures. I hope that this will also help address the query raised in Comment 3. I also added a cartoon drawing to Fig. 5 to clearly present the experimental procedures.
